# Medical Heroism

**Published:** 1859-09

**Authors:** 


MEDICAL HEROISM.—Such is the title of an address delivered by Dr. John Bell, before the Philadelphia County Medical Society, on retiring from the office of President. It is characterized by great learning, as are all the productions of this distinguished author; and is one of the most interesting discourses which it has ever fallen to our lot to read. We do not recollect ever before to have seen the subject of medical heroism discussed with half so much ability. Our limits will permit only one extract:—

"The name of Howard is everywhere celebrated, and praised in terms of warm gratitude, as the reformer of prison abuses and prison cruelties. It has obtained a place in the history of the world's progress. The name of Pinel is not, I am afraid, familiar even to the medical world; and it is still less to the world at large, as that of a physician, who, both by personal services and earnest teaching, brought about a reform in the management and discipline of asylums for the insane, which may now be properly regarded one of the strongest proofs of advanced civilization. If a proper sympathy and sentiment for humanity and justice have been enlisted by the benevolent Englishman, in what light ought we to regard the services of the equally benevolent Frenchman, who reminded men of their duties to the Providence stricken, but irresponsible insane? Excuse might be found for vindictive harshness to the criminal who has made war on society; but where is the extenuation for more deliberate cruelty, practiced so long and so generally on those unfortunate beings, bereft of their reason, many of whom, but a short time before, had been the delight of the social circle, and cherished members of the family?

"When we think of the old Bedlams and hospitals for the insane, in which not only the raving maniac, but the melancholy monomaniac was confined, and in which the only sounds were those of the clanging chain, the echoing lash, and the mingled cries and vociferations of the brutal keepers and infuriated inmates; and then look abroad over the better portions of the civilized world, including our own favored land, and see the many noble edifices erected for the reception and treatment of this class of unfortunate fellow-beings, we feel that we live in an age not only of progress, but of real improvement; one in which humanizing influences are more active and diffused than they ever were before. The contrast between the present and the past in this particular, while it should prompt all to the liveliest manifestations of gratitude, ought, undoubtedly, to find a place in general history, in which proper credit would be awarded to our profession, so many members of which have imitated, in their official position as superintendents of insane asylums, the noble example set by Pinel at the Bicêtre and the Salpêtrière.

"That was indeed a critical moment in the life of Pinel, and in the history of benevolent trials for the mitigation of human suffering, when he resolved to test the correctness of his principles of non-restraint, by holding direct personal intercourse with a violent maniac, whose chains and fetters he had previously directed to be removed. The trial was entirely successful.

After an eager gaze and a movement, as if preparatory to a tiger-like spring on his visitor, who had just entered his cell, the unfortunate being saw eyes beaming with kindness and placid features, expressing benignity and good-will. Soon his own countenance underwent a change; the mere brute was once again a human being; and when the tones of affectionate inquiry reached his ear, and the hand of greeting was extended toward him, he could only answer and reciprocate by shedding tears, the fountains of which had long been dried up by the fiery furnace of maddened feelings, wrought to fury by angry menace and brutal punishment. From this moment the cure of the poor maniac, which had been before regarded as hopeless, was begun, and terminated in entire restoration to health and reason."

